# Anserine (beta-alanyl-3-methyl-L-histidine) improves neurovascular-unit dysfunction and spatial memory in aged AβPPswe/PSEN1dE9 Alzheimer’s-model mice

**DOI:** 10.1038/s41598-017-12785-7

**Published:** 2017-10-03

**Authors:** Jun Kaneko, Akiko Enya, Kota Enomoto, Qiong Ding, Tatsuhiro Hisatsune

**Affiliations:** Department of Integrated Biosciences, The University of Tokyo, Kashiwa, Japan

## Abstract

Anserine/carnosine supplementation improves cerebral blood flow and verbal episodic memory in elderly people, as we previously reported. Anserine’s buffering activity is superior to that of carnosine at neutral pH. In human sera, carnosine but not anserine is rapidly cleaved by carnosinase, limiting its effectiveness. This study examined the effects of anserine on AβPPswe/PSEN1dE9 Alzheimer’s disease (AD) model mice over 18-months old, an age at which these mice exhibit detectable memory deficits. We found that 8 weeks of anserine treatment completely recovered the memory deficits, improved pericyte coverage on endothelial cells in the brain, and diminished chronic glial neuroinflammatory reactions in these mice. These results suggest that anserine (beta-alanyl-3-methyl-L-histidine) supplementation improved memory functions in AD-model mice by exerting a protective effect on the neurovascular units, which are composed of endothelial cells, pericytes, and supporting glial cells.

## Introduction

Histidine-containing dipeptides such as carnosine and anserine act as biochemical buffers, chelators, antioxidants, and anti-glycation agents^1–4^. Carnosine, an endogenous dipeptide consisting of beta-alanine and L-histidine, is found primarily in excitable cells such as skeletal, cardiac, and smooth-muscle cells, where it is present in the millimolar range^4^. Carnosine treatment is beneficial in animal models of brain ischemia and diabetes-induced nephropathy^4^. Carnosine also prevents neurovascular damage and memory deficits in a transgenic Alzheimer’s disease (AD) model mouse fed a high-fat diet^5^.

Carnosine is cleaved by carnosinase into beta-alanine and histidine. In rodents, the serum carnosinase activity is low, so carnosine treatment elevates the plasma carnosine level^4^. In humans, however, a high serum carnosinase activity limits the efficacy of carnosine supplements^6^. Anserine (beta-alanyl-3-methyl-L-histidine), a methylated form of carnosine, is a natural carnosine derivative that is not cleaved by serum carnosinase. Anserine was first discovered in goose muscle in 1929^7^, and was named after this extraction (*anser* is Latin for goose). At present, anserine alone is not commercially available as a food or medication; therefore, we and Szcześniak *et al*.^8,9^ used anserine and carnosine (3:1) from chicken meat for anserine supplementation. Anserine, which is water-soluble, is found at high levels in the muscles of salmon and tuna, which are migratory fish. There are few reports on the biomedical function of anserine, probably due to the low availability of pure anserine compared to carnosine, but anserine is reported to be bioactive and to have both buffering and anti-inflammatory activities^10,11^.

AD is a prototypical neurodegenerative dementia associated with old age^12–14^. Recent studies have revealed that brain blood-vessel dysfunction is part of the AD pathogenesis^15–19^. In 2008, Zlokovic *et al*. reported a previously unknown feature of AD in humans and in AD-model mice, namely, that microvascular defects in blood vessels in the brain impair the clearance of neurotoxic molecules^15^. In line with these observations, we previously demonstrated that anserine/carnosine supplementation increases blood flow in part of the brain and ameliorates memory deficits in healthy elderly people^8^. Similarly, Szcześniak *et al*.^9^ suggested that anserine and carnosine supplements might improve physical and cognitive functions in elderly people.

This study was conducted to determine whether anserine treatment could improve neurovascular-unit and cognitive functions in aged AD-model mice. Neurovascular units in the brain are composed of vascular cells (endothelium, pericytes, and vascular smooth-muscle cells) and supported glial cells, including astrocytes and microglia. Recent studies showed that pericytes play pivotal roles in the neurovascular unit and in the pathogenesis of AD^16,19,20^. Here we found that anserine treatment reversed the spatial learning and memory deficits in AD mice. Our detailed examination of the brain tissue in these mice indicated that anserine’s effect was due to a protective activity against pericyte degeneration.

## Results

### Anserine treatment improved cognitive impairment in aged AD-model mice

**Figure 1:**
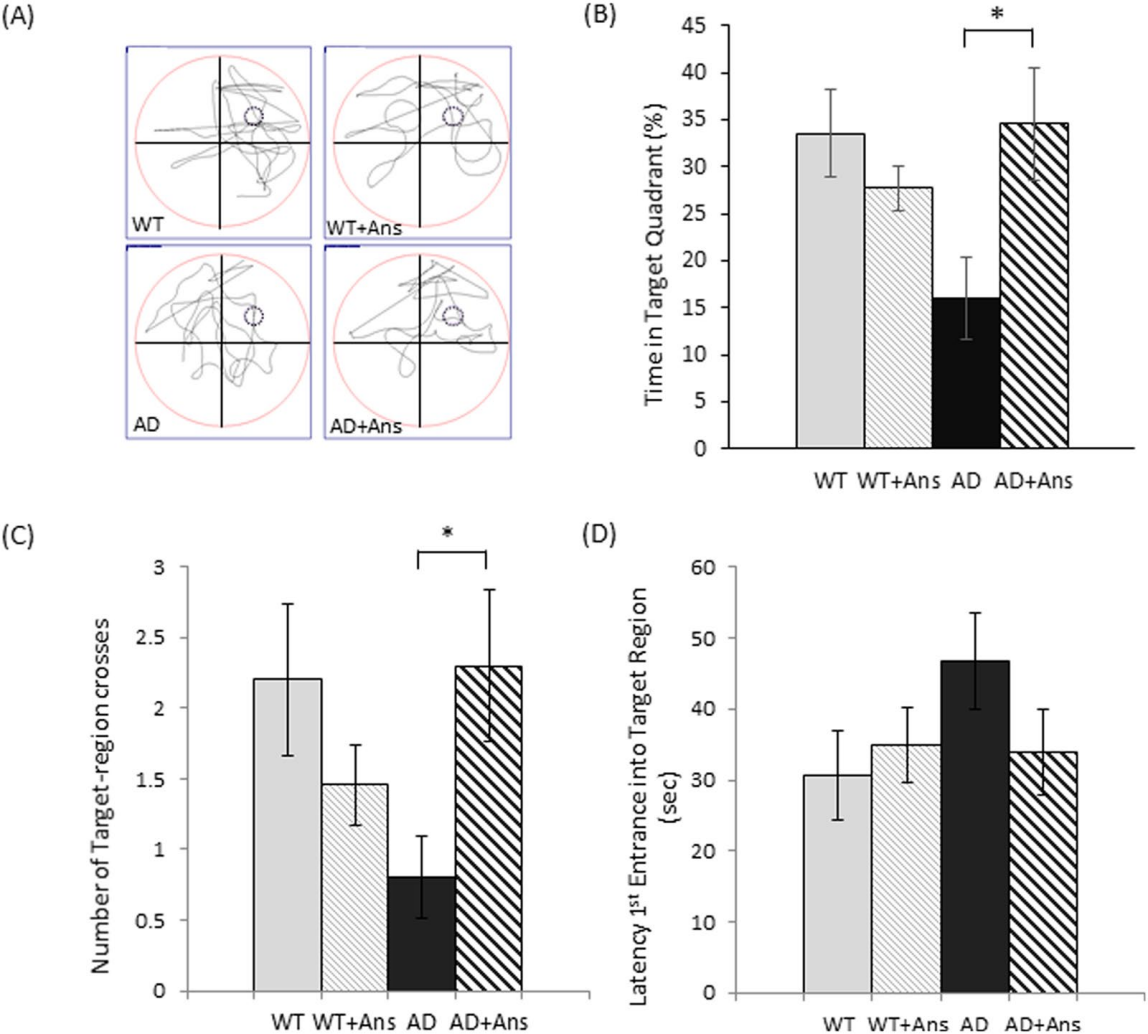
Anserine improved cognitive deficits in aged AD-model mice. Cognitive function in aged, anserine-treated (+Ans) or untreated AD and WT (control) mice was assessed by a Morris Water Maze probe test. (**A**) A representative swimming trajectory from each group during the probe test. (**B**) Average time spent in the target quadrant. Data were analyzed by a two-way ANOVA (Interaction of Genotype × Treatment F[1,37] = 7.494, *p* = 0.0095). After a Holm-Sidak *post-hoc* test, we observed a significant difference between AD and AD + Ans (t[2.914], *p < 0.05). (**C**) Number of crossings of the target region. Data were analyzed by a two-way ANOVA (Interaction of Genotype × Treatment F[1,37] = 7.015, *p* = 0.0118). After a Holm-Sidak *post-hoc* test, we observed a significant difference between AD and AD + Ans (t[2.474], *p < 0.05). (**D**) Latency of the first entrance into the target region (Interaction of Genotype × Treatment F[1,37] = 3.285, *p* = 0.0780). After a Holm-Sidak *post-hoc* test, we observed a tendency toward a difference between AD and AD + Ans (t[2.041]). WT, n = 10; WT + Ans, n = 11; AD, n = 10; AD + Ans, n = 10. Mean ± SEM.

In a pilot experiment, we used 6-month-old AD model mice fed a high-fat diet for 8 weeks, and then performed two memory tests: the Morris water maze (MWM) and contextual fear conditioning test (Supplementary Fig. 1). We found that anserine treatment significantly suppressed the decline in cognitive function in these two tests. These pilot experiments revealed that anserine treatment was beneficial for preserving the memory performance of this AD mouse fed a high-fat diet, as reported previously for carnosine supplementation^5^. However, using mice fed a high-fat diet, it is possible that a diabetes-related pathophysiological alteration plays a role in the effect on memory^5^. Therefore, in this study, to assess whether anserine treatment protected against the detrimental alteration in AD model mice without any diabetes-related alterations, we used 18-month-old AD model mice, in which the decline in cognitive function is detectable with a relatively small sample size (fewer than 10 mice per group). We compared the memory function in untreated and anserine-treated AD-model mice (AD and AD+Ans). We also used data from untreated and anserine-treated wild-type groups (WT and WT +Ans), for reference. After 8 weeks of anserine treatment, the mice underwent the MWM test. Figure 1A shows representative trace data from each group in the probe test. Compared to WT mice, untreated AD mice spent less time in the target quadrant. Notably, anserine-treated AD mice (AD+Ans) spent significantly more time than untreated AD mice in the target quadrant (*p < 0.05) (Fig. 1B). There was a significant difference in the number of times the mice crossed the target region between the two AD groups (Fig. 1C; AD and AD+Ans, *p* < 0.05), and a tendency toward a decreased latency in entering the target region in the AD+Ans group compared to the AD group (Fig. 1D). We also evaluated the effect of anserine treatment on memory improvement using a novel object location test, and observed a similar effect of anserine (Supplementary Fig. 2). These results demonstrated that anserine treatment improved the spatial-memory impairment exhibited by AD-model mice.

### Anserine treatment improved neurovascular-unit function

**Figure 2:**
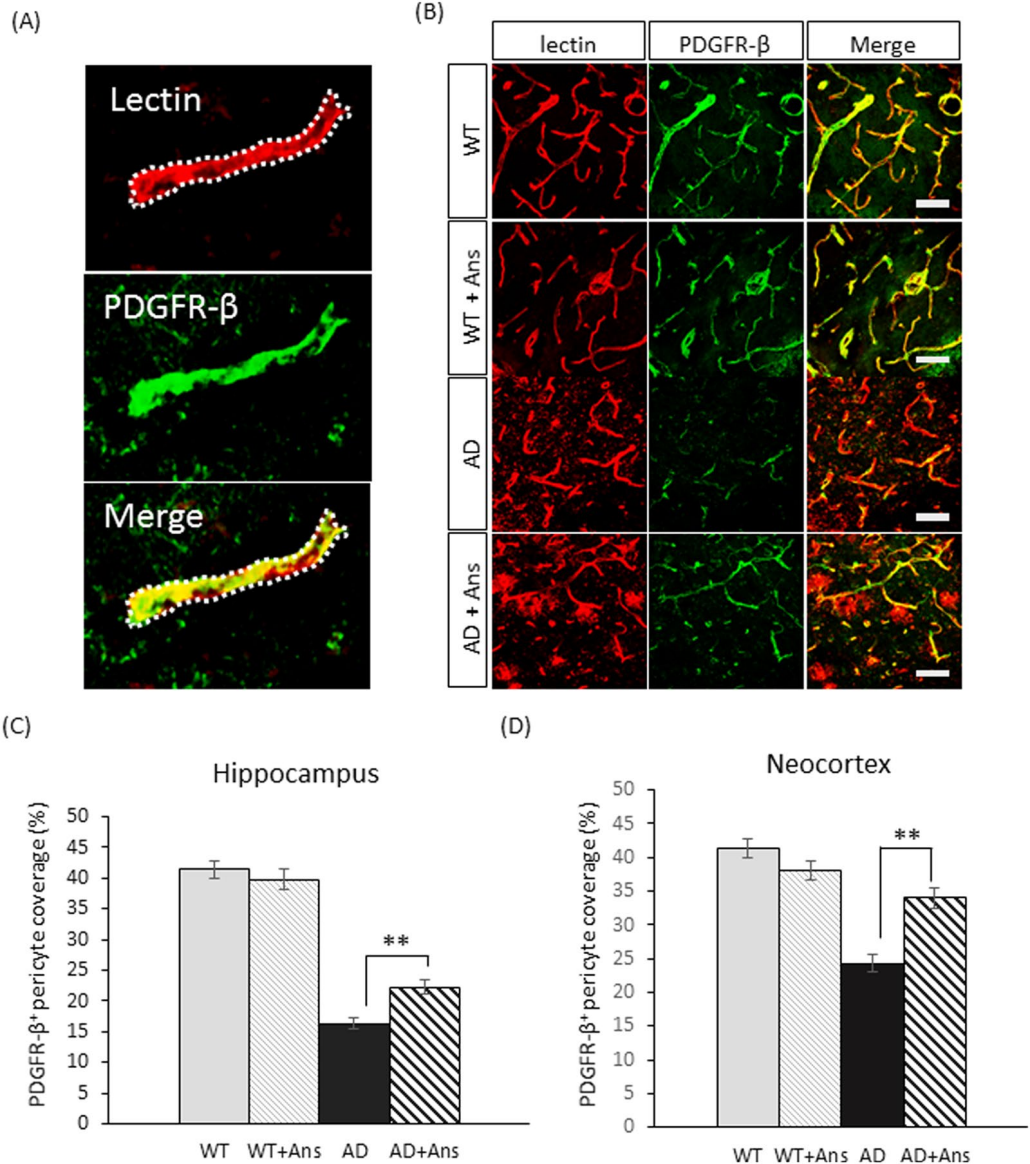
Anserine improved the reduced pericyte coverage in the hippocampus of aged AD-model mice. (**A**) A representative microscopic image of lectin^+^ endothelial cells (red) and PDGFR-β^+^ pericytes (green). An ImageJ plugin^5^ was used to outline the lectin-positive area as a region of interest (ROI). Pericyte coverage was determined as the percentage of PDGFR-β–positive pixels within the ROI. The rate of PDGFR-β coverage on endothelial cells was calculated automatically with a custom ImageJ plugin. (**B**) Images of histochemically stained PDGFR-β^+^ pericytes and lectin^+^ endothelial cells in blood vessels in the hippocampus of untreated and anserine-treated (+Ans) WT and AD mice. Scale bar: 50 μm. (**C**) The coverage rate of PDGFR-β^+^ pericytes on lectin^+^ vascular endothelial cells in the hippocampus was calculated as described in A. Pericyte coverage was increased in the hippocampus of AD-model mice after anserine treatment. Data were analyzed by a two-way ANOVA (Interaction of Genotype × Treatment F[1,366] = 8.680, *p* = 0.0034). After a Holm-Sidak *post-hoc* test, we observed a significant difference between AD and AD+Ans (t[3.428], **p < 0.01). (WT, N = 3, n = 88; WT+Ans, N = 3, n = 78; AD N = 3, n = 117; AD+Ans, N = 3, n = 87). Mean ± SEM. (**D**) The coverage rate of PDGFR-β^+^ pericytes on lectin^+^ vascular endothelial cells in the neocortex was calculated as described in A. Data were analyzed by a two-way ANOVA (Interaction of Genotype × Treatment F[1,264] = 19.98, *p* < 0.0001). After a Holm-Sidak *post-hoc* test, we observed a significant difference between AD and AD + Ans (t[5.026], **p < 0.01). WT, N = 3, n = 50; WT + Ans, N = 3, n = 72; AD, N = 3, n = 74; AD + Ans, N = 3, n = 72. Mean ± SEM.

An important indicator of neurovascular-unit function is the rate at which pericytes, a key component of the neurovascular unit, cover brain capillaries. We analyzed images of stained brain tissue by outlining the edges of lectin^+^ endothelial cells, superimposing a PDGFR-β-stained image on it, and calculating the area of PDGFR-β^+^ pericytes overlapping the outlines as the rate of pericyte coverage (Fig. 2A). Figure 2B and C show that pericyte coverage in the hippocampus was decreased in both the untreated and anserine-treated AD groups compared to the WT groups, but that this degeneration in the AD group was significantly improved by anserine treatment (**p < 0.01). Pericyte coverage was also decreased in the cortex of the AD mice (Fig. 2D), and again, this degeneration was significantly improved by anserine treatment (**p < 0.01). Thus, pericyte coverage was reduced in both the hippocampus and the cortex of the aged AD-model mice, and this reduction was partially recovered by the 8-week course of anserine treatment.

**Figure 3:**
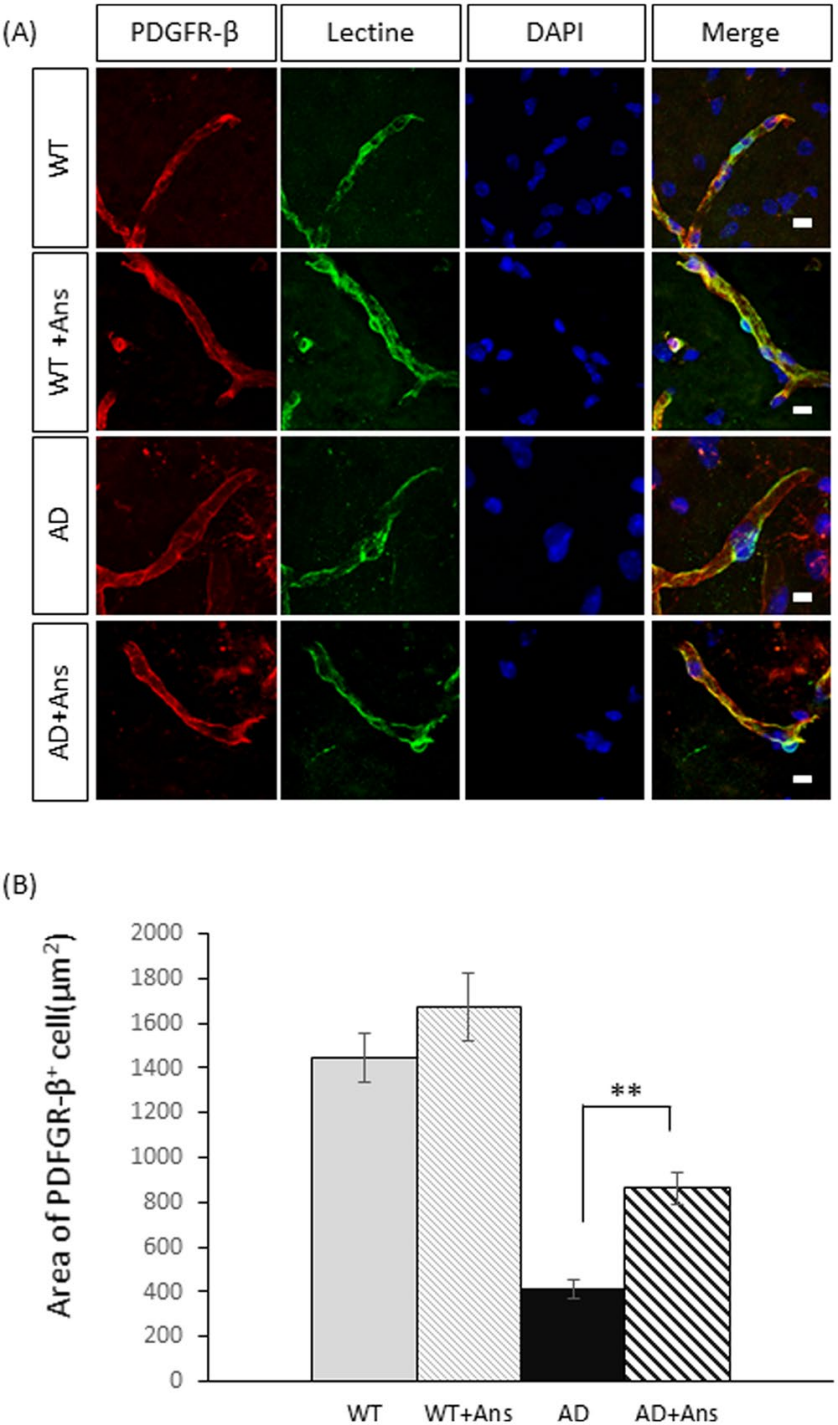
Anserine reduced the pericyte shrinkage in aged AD-model mice. (**A**) A confocal-microscope image used to assess pericyte shrinkage on blood vessels in the brain, showing PDGFR-β^+^ pericytes (green) and lectin^+^ endothelial cells (red); nuclei were stained with DAPI (blue). (**B**) Average area of individual pericytes in each group. Data were analyzed by a two-way ANOVA (Treatment F[1,125] = 10.80, *p* = 0.0013). After a Holm-Sidak *post-hoc* test, we observed a significant difference between AD and AD+Ans (t[2.993], **p < 0.01). WT, N = 3, n = 38; WT+Ans, N = 3, n = 31; AD, N = 3, n = 32; AD + Ans, N = 3, n = 28. Mean ± SEM.

**Figure 4:**
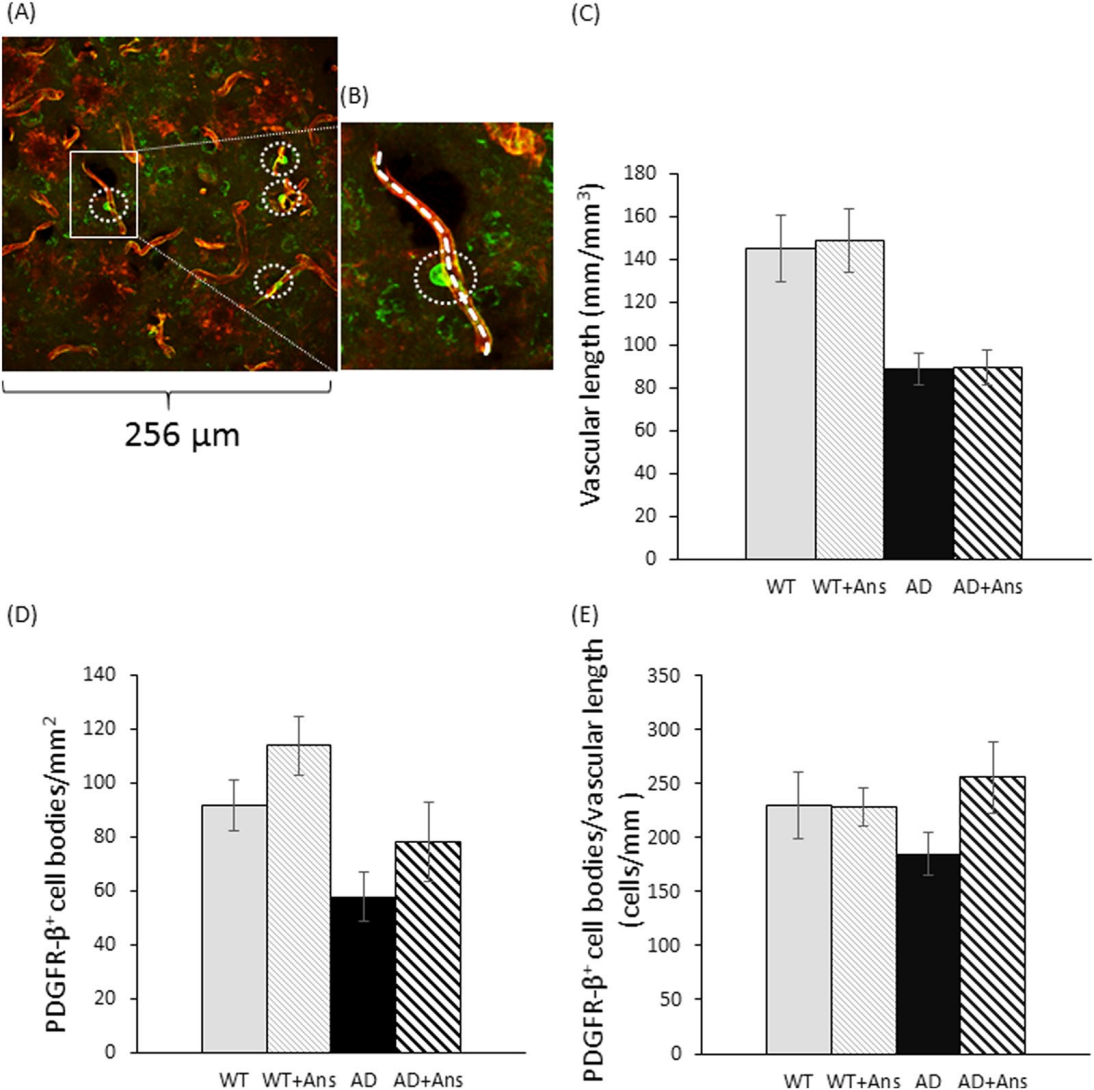
Anserine did not affect the number of pericytes per unit blood vessel in the brain of aged AD-model mice. (**A**) A representative confocal microscopy image of a PDGFR-β^+^ pericyte cell body (green) on lectin^+^ vascular endothelial cells (red). (**B**) Magnified view of the white rectangle in panel A. Using ImageJ, a dotted line was drawn along the lectin^+^ endothelial cells, and its length was calculated as the vascular length. (**C**) Vascular length was calculated for untreated and anserine-treated (+Ans) AD mice. The total length of lectin^+^ endothelial cells per hippocampal area is shown. The total blood-vessel length was decreased in AD-model mice compared to WT mice, as analyzed by a two-way ANOVA (Genotype F[1,391] = 25.23, *p* < 0.0001). The treatment did not cause a difference (Treatment F[1,391] = 0.0039, *p* = 0.8431). WT, N = 3, n = 95; WT+Ans, N = 3, n = 90; AD, N = 3, n = 110; AD + Ans, N = 3, n = 100. Mean ± SEM. (**D**) Number of PDGFR-β^+^ cell bodies per mm^2^ of tissue section in the hippocampus. The number of pericytes was decreased in the AD compared to the WT hippocampus (Genotype F[1,32] = 9.538, *p* = 0.0041), but did not change due to treatment (Treatment F[1,32] = 3.546, *p* = 0.0688) (WT, N = 3, n = 9; WT+Ans, N = 3, n = 9; AD, N = 3, n = 9; AD+Ans, N = 3, n = 9). Mean ± SEM. (**E**) Data obtained as in (**C**) and (**D**) were used to calculate the number of PDGFR-β^+^ cell bodies per vascular length for each group. Anserine treatment did not alter the number of pericytes per vascular length (Treatment F[1,8] = 1.725, *p* = 0.2255). Mean ± SEM.

We also compared the average area per PDGFR-β^+^ pericyte (i.e., the cell size) and the number of pericytes per unit of blood capillaries in untreated and anserine-treated AD and WT mice. As shown in Fig. 3, the average area per pericyte was significantly reduced in untreated AD mice (**p < 0.01), and this reduction was significantly recovered in anserine-treated AD mice (**p < 0.01). Next, to count the number of pericytes per unit of blood capillaries, we measured the average blood-vessel length per unit area in the hippocampus for each of the four groups. Figure 4A shows a representative confocal microscopic image of a PDGFR-β^+^ pericyte cell body (green) on lectin^+^ vascular endothelial cells (red). We estimated the total length of the vasculature in the image by measuring each length of lectin^+^ endothelial cells (Fig. 4B). We compared the length of brain vasculature per hippocampal area between the groups (Fig. 4C). The total blood-vessel length was decreased in AD-model mice compared to WT, and this decrease was not prevented by anserine treatment. We also found an approximately 35% reduction in lectin^+^ blood vessels in the hippocampus of the AD groups compared to the WT groups. We determined the number of PDGFR-β^+^ (pericyte) cell bodies per hippocampal area (Fig. 4D), and found an approximately 35% reduction in the number of pericytes per unit area in the AD groups. Notably, however, the number of pericytes per unit of blood capillaries was equivalent in the AD and WT groups (Fig. 4E). Considered together, these results indicated that the area covered by pericytes in AD mice decreased due to individual pericyte shrinkage and that anserine recovered this shrinkage, thereby restoring the pericyte coverage rate and contributing to the functional recovery of neurovascular units in AD-model mice.

### Anserine did not affect Aβ accumulation in AD-model mice

**Figure 5:**
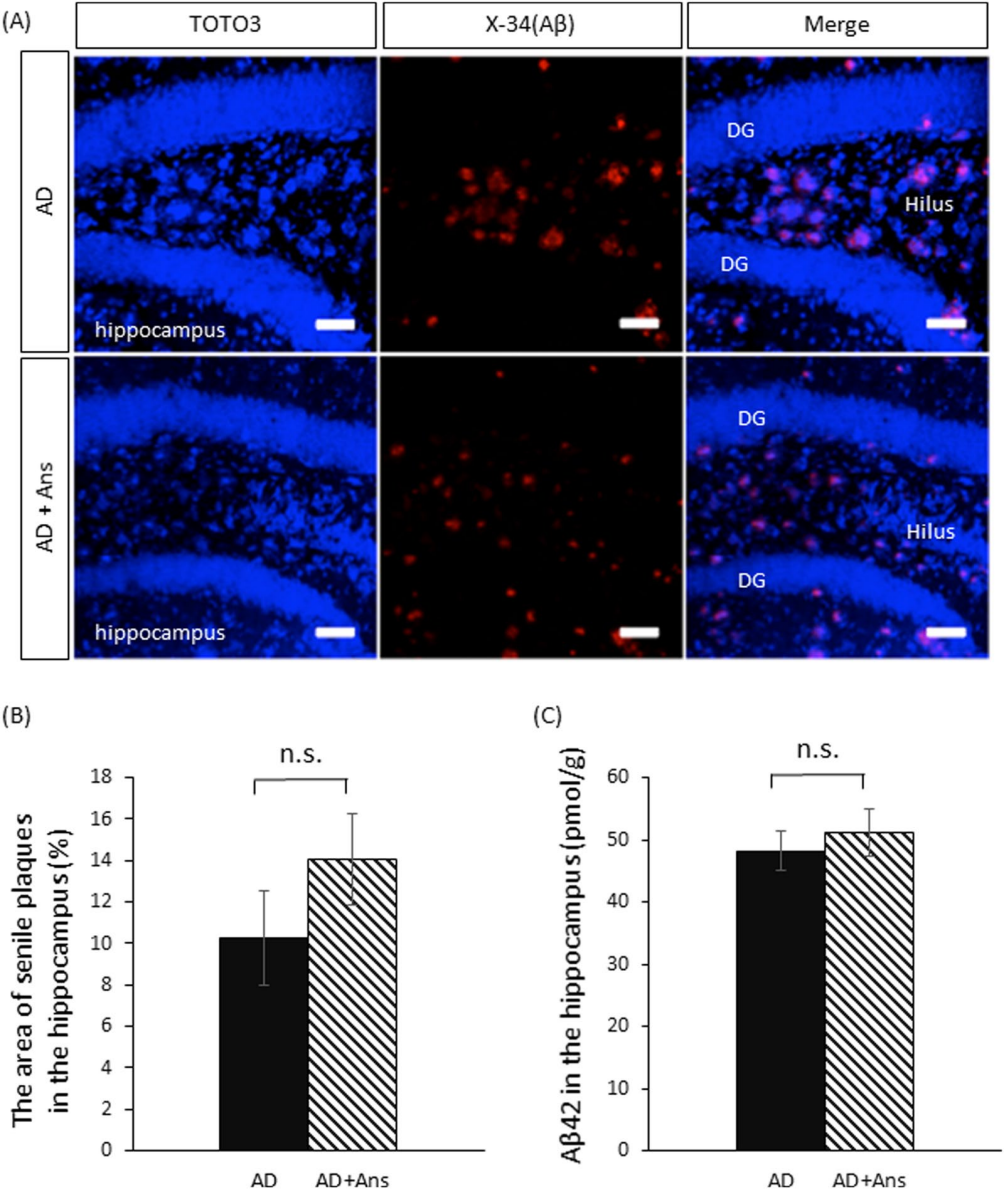
Anserine did not affect the Aβ accumulation in aged AD-model mice. (**A**) Confocal-microscopy images of senile plaques stained by X-34 (red) in the hippocampus of aged AD-model mice; neuron nuclei were stained by TOTO3 (blue). DG, dentate gyrus. Scale bars: 50 µm. (**B**) Percentage of Aβ^+^ area (stained by X-34, red) in the hippocampus. Anserine treatment (+Ans) did not decrease the amount of senile plaque. (**C**) Aβ42 in the AD hippocampus, determined by ELISA, was not decreased by anserine treatment. (Histochemical staining: AD, n = 3; AD+Ans, n = 3, *p* = 0.30, Student’s *t* test. ELISA: AD, n = 7; AD+Ans, n = 9, *p* = 0.57, Student’s *t-*test.). Mean ± SEM. n.s., not. significant.

To determine the effect of anserine on the formation of senile plaques and the accumulation of the toxic Aβ peptide Aβ1-42, we analyzed untreated and anserine-treated AD mice by ELISA and by histochemical staining with X-34, which labels senile plaques. There was no difference in the appearance of senile plaques (Fig. 5A,B) or in the accumulation of Aβ1-42 in the hippocampus of anserine-treated versus control AD-model mice (Fig. 5C). Thus, anserine did not appear to affect the senile-plaque formation or Aβ1-42 accumulation (*p* = 0.30, *p* = 0.57, respectively, Student’s t-test).

### Anserine suppressed glial inflammatory reactions

**Figure 6:**
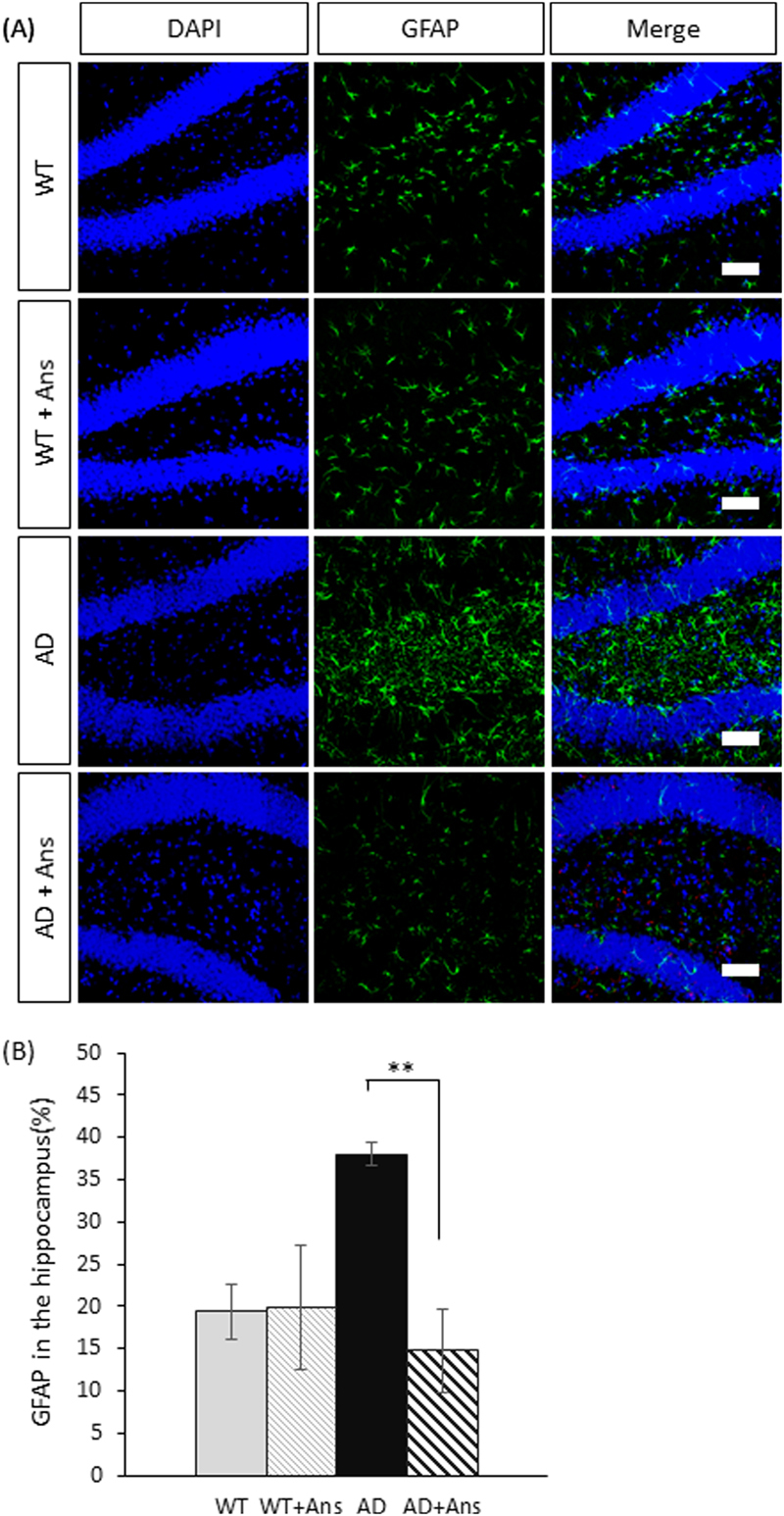
Anserine suppressed glial inflammation in aged AD-model mice. (**A**) Confocal-microscopy images of activated inflammatory astrocytes stained with anti-GFAP (green). DG, dentate gyrus. Scale bars: 50 µm. (**B**) Average area of GFAP^+^ astrocytes in the hippocampus of aged untreated or anserine-treated (+Ans) AD-model mice. GFAP expression was significantly elevated in the AD group, and this elevation was completely blocked by anserine treatment. Data were analyzed by a two-way ANOVA (Interaction of Genotype × Treatment F[1,8] = 10.18, *p* = 0.0128). After a Holm-Sidak *post-hoc* test, we observed a significant difference between AD and AD+Ans (t[4.406], **p < 0.01). WT, n = 3; WT+Ans, n = 3; AD, n = 3; AD+Ans, n = 3. Mean ± SEM. analysis.

**Figure 7:**
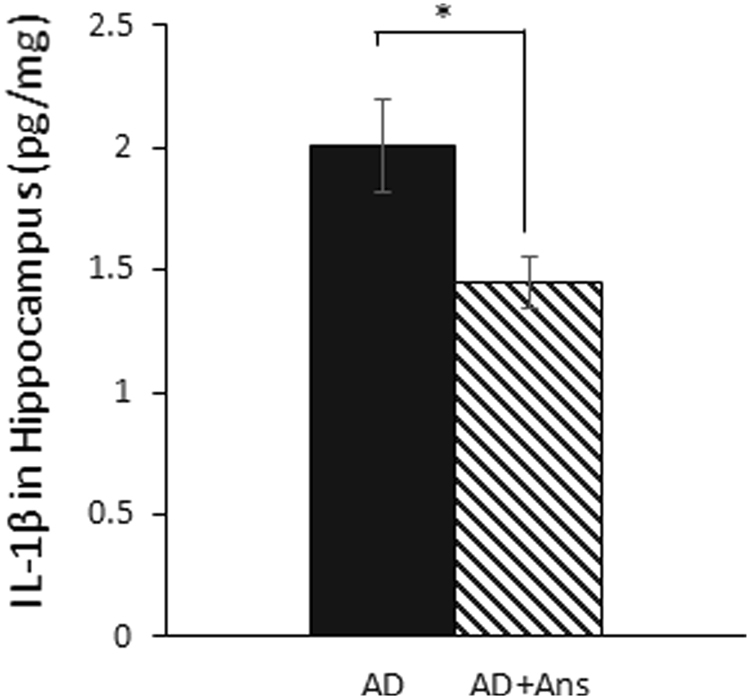
Anserine reduced IL-1β in the hippocampus of aged AD-model mice. The inflammatory cytokine interleukin 1β (IL-1β) was measured in the hippocampus of untreated and anserine-treated (+Ans) AD mice. Anserine decreased the amount of IL-1β in AD-model mice (Mean ± SEM; AD, n = 7; AD+Ans, n = 9, *p* = 0.02, Student’s *t-*test.). In a separate experiment, we measured the amount of IL-1β in the wild-type groups (control and anserine treated). The average amount in both groups (WT and WT+Ans) was below 1 pg/ml.

To evaluate the degree of glial neuroinflammatory reactions in the hippocampus of AD-model mice, we performed histochemical staining for activated astrocytes. We found that the area expressing GFAP, which marks activated astrocytes, was larger in the hippocampus of the AD group compared to the WT group, and that astrocyte activation was significantly suppressed in the anserine-treated AD group (**p < 0.01) (Fig. 6). In addition, IL-1β, which increases in the brain as inflammation progresses, was found in the hippocampus at significantly lower levels in anserine-treated than in untreated AD mice (*p* = 0.02, Student’s t-test) (Fig. 7), indicating that anserine suppressed glial neuroinflammatory reactions in the AD brain. These results suggested that the 8-week course of anserine treatment suppressed hippocampal inflammation in these aged AD-model mice.

## Discussion

The present study demonstrated that anserine treatment improved the spatial-memory performance in aged AD-model mice. Anserine (10 mg/mouse/day) was administered to AD transgenic mice over 18 months of age, after the onset of AD. O’Leary and Brown reported that the transgenic AD-model mice used in this study show measurable cognitive impairment at 16 months of age^21^. We have also observed memory decline in 12-month-old AD-model mice as evaluated by the MWM test (J.K and T.H. unpublished observation). Here we found that an eight-week course of anserine treatment improved the decline in spatial-memory function of AD-model mice over 18 months old as evaluated by the MWM (Fig. 1) and novel object location (Supplementary Fig. 2) tests. This study is the first to demonstrate that anserine treatment improves the spatial memory performance of AD model mice over 18 months old, at a stage when these mice exhibit detectable deficits in memory performance.

**Figure 8:**
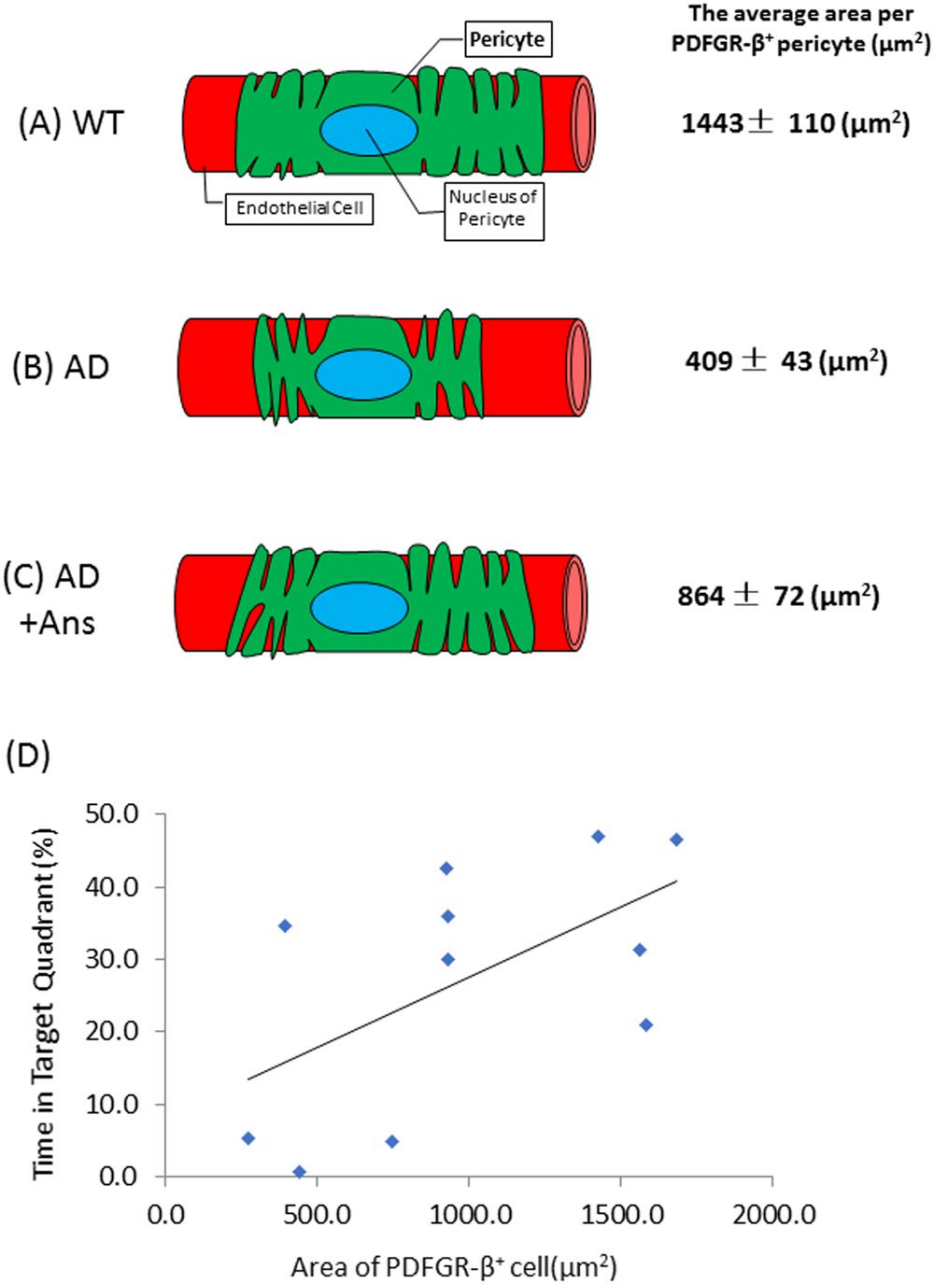
Schematic illustration of the effect of anserine on pericytes in aged AD-model mice. The area covered by individual pericytes shrank in AD mice (**B** versus **A**), and anserine reversed this shrinkage (**C** versus **B**), thereby restoring the pericyte coverage and contributing to the functional recovery of neurovascular units. The average area per PDFGR-β+ pericyte (µm^2^) was calculated in Fig. 3. (**D**) Correlation between the average area per PDFGR-β+ pericyte (µm^2^) and the score of memory performance in the MWM, using the data described in Fig. 3B and Fig. 1B, analyzed by the Pearson correlation coefficient (Correlation coefficient = 0.58, *p < 0.05).

In the present study, we further found that anserine treatment ameliorated pericyte degeneration and glial neuroinflammation in the AD mouse brain. Blood-vessel damage is a common feature in the AD brain^17,19^. Pericytes, which lie on the capillary surface, are important for neurovascular-unit function. Anserine treatment increased the rate of pericyte coverage by reversing the pericyte shrinkage observed in aged AD-model mice. A schematic illustration of this mechanism is shown in Fig. 8A–C. We also performed a correlation analysis using our data for memory performance (Fig. 1B) and pericyte coverage (Fig. 3B), and found a significant correlation between these two parameters (Fig. 8D). To our knowledge, this is the first report to demonstrate the recovery of brain pericyte function, indicated by morphological recovery, which may causally relate to the recovery of memory performance in aged-AD model mice. We recently performed a human randomized control trial (RCT) study, in which elderly volunteers took anserine/carnosine supplementation (ACS) for 12 months, and found that the supplementation preserved blood flow in the prefrontal brain and improved the episodic memory performance of these subjects^22^. In addition, in a separate 12-week RCT, we observed that cognitive functions were preserved by ACS in patients with mild cognitive impairment (T.H. submitted separately). Taken together, these studies suggest that the preservation of brain blood flow by anserine/carnosine supplementation is linked to the preservation of memory performance, in elderly people as well as in AD model mice.

Both carnosine and anserine ameliorate the cognitive decline seen in AD-model mice, as seen in our present and previous studies^5^. Carnosine and anserine are both histidine-containing dipeptides with buffering ability in a neutral-pH range. In addition to their dipeptide amino and carboxyl residues, carnosine and anserine also have a second pKa within a neutral pH range, with an estimated pK of 6.83 and 7.04, respectively^1,23^. Pericytes, like skeletal muscle cells, are derived from mesenchymal stem cells^24,25^, and the pericyte cell body, like skeletal muscle cells, contains actomyosin fibers. From these observations, it is reasonable to speculate that anserine might reverse the AD-related degeneration of brain pericytes through a mechanism similar to the compensation of skeletal-muscle tissue, via its strong buffering activity^4^. Thus, anserine might protect neurovascular-unit function by improving pericyte function, thereby suppressing neuroinflammation and restoring cognitive function in AD-model mice. In the presence of brain microvascular abnormalities and leakage, neuroinflammation and oxidative stress accelerate the production of neurotoxic molecules such as chemokines, which interfere with the normal neural transmission in the brain^26–29^. As an alternative mechanism, it is possible that anserine treatment decreases neuroinflammation by suppressing glial inflammatory reactions, thereby protecting the neuro-vascular unit from damage and decreasing pericyte degeneration. Further study is needed to elucidate the mechanism by which memory is preserved by anserine treatment in AD model mice.

Anserine treatment significantly suppressed neurovascular-unit dysfunction and neuroinflammatory reactions in AD model mice. Accelerated pericyte degeneration is known to cause blood-brain barrier breakdown and to lead to neuronal degeneration in AD-model mice^18^ and in the brain of AD patients^19^. In the AD-model mouse, glial cells close to Aβ accumulations are activated, and the activated glial cells release inflammatory cytokines and further promote neuroinflammation^30^. As inflammation increases, memory function deteriorates^31^. Here we observed that the number of activated inflammatory astrocytes in the hippocampus decreased significantly after anserine treatment. The level of the inflammatory cytokine IL-1β, which is released by activated glial cells, also decreased. We previously showed that a choline esterase inhibitor, rivastigmine, suppresses astroglial activation, and decreases the amount of IL-1β in AD model mice, without affecting the senile plaque load^31^. In the present study, we found that anserine treatment suppressed the astroglial activation, but not the senile plaque load (Fig. 5). The use of anti-inflammatory medication before the onset of clinical symptoms is associated with reduced cognitive decline in AD^32,33^. In the AD-model mouse, dysfunctional mitochondria in activated astrocytes are a source of neuroinflammatory reactions^34^. Our observations suggest that anserine treatment inhibited hippocampal inflammation and the activation of inflammatory glial cells, particularly of astrocytes, in the AD mice. Our results indicate that anserine treatment can reverse some AD-induced decline in spatial cognitive function in aged AD-model mice, probably by ameliorating pericyte degeneration and neuroinflammation in the brain of these mice.

## Methods

### Animals

B6C3-Tg (APPswe/PSEN1dE9) 85Dbo/J AD-model mice were purchased from Jackson Laboratories (Bar Harbor, Maine, USA), and subsequent generations were bred in our laboratory. These AD-model mice express the Swedish variation of the phenotype, presenting both a chimeric human APP transgene (Mo/HuApp695swe) and a human PS1 transgene (missing exon 9)^35^. To produce WT and transgenic mice of a similar background, the transgenic mice were interbred with WT mice of the same lineage (B6C3, purchased from Jackson Laboratories). Successive generations of WT mice were also bred in our laboratory. To obtain a sample consistent with the occurrence of AD in human populations, male and female mice over 18 months old were used in this study. The mice were bred in plastic cages in groups of up to five depending on the genotype and age. The animals were maintained at 22 °C with a 12-h light–dark cycle. All animal procedures and experiments in this study were approved by the ethical committee of the University of Tokyo and were conducted according to the guidelines for animal experimentation required by the University of Tokyo.

### Anserine treatment

To obtain a large amount of anserine, free of carnosine, for long-term supplementation in mice, we purified anserine from salmon muscle, which contains anserine but not carnosine^4^ by two-step chromatography (purity > 93%, Tokai Bussan, Tokyo, Japan). Anserine-treated mice were maintained on a steady dosage of anserine diluted in autoclaved drinking water, at a concentration of 2.0 g/L (equivalent to 10 mg/mouse), for 8 weeks, and the experiments ended at the end of the treatment period. Control animals were maintained on regular autoclaved drinking water. All animals had access to drinking water, regular or anserine-treated, ad libitum.

### Memory tests

Spatial learning and memory were assessed in untreated and anserine-treated AD and WT mice by MWM testing for 6 consecutive days. Each mouse was habituated to the experimenter for 5 min per day for 3 consecutive days prior to beginning the MWM testing. The tests were conducted in a dimly lit circular pool (120-cm diameter, 35-cm deep) filled with opaque water (23 °C ± 2 °C) and surrounded by visual cues. The starting locations were semi-randomly chosen from northeast (NE), southeast (SE), southwest (SW), or northwest (NW), and the platform was placed in the middle of the NE quadrant. The tests were conducted as described previously, with some modifications^31^. Briefly, the MWM sessions on days 1–5 were conducted as an acquisition test to evaluate learning. After the initial training, a probe test was conducted on day 6 (24 h after the last training session) to assess memory function. The platform was removed. The mouse was allowed to swim for 60 s starting from the W side of the pool (single-probe trial), and the time the mouse spent in the target quadrant (NE), where the platform was previously located during the learning session, was measured. All data were recorded and analyzed with the SMART video tracking system (Panlab, Barcelona, Spain). The contextual fear conditioning test was carried out as described before^5,31^. In brief, we used a fear conditioning apparatus made by Med Associates Inc. that consists of a metal-acrylic box connected to a video camera, which enabled automated recording and analysis of the experiments. The novel object location (NOL) task was based on Xiong *et al*.^36^ with some modifications. In brief, the NOL task was performed across four days: two days for habituation, and the following two days for testing (See Supplementary Materials and Methods for details).

### Sample processing

Animals were sacrificed and transcardially perfused with phosphate-buffered saline (PBS) to remove blood and with 4% paraformaldehyde to fix the brain tissue. Brain samples were postfixed in 4% paraformaldehyde for 24 h, incubated in 30% sucrose/PBS for 3 days, and then embedded in Tissue-Tek OCT compound (Sakura Finetek, Japan) and frozen at −80 °C. Samples were sliced into 40-µm-thick coronal sections with a Cryostat (Microm, Germany) while kept at −20 °C. Hippocampal slices were kept in a cryoprotectant solution and stored at −30 °C.

### Histochemical staining

To calculate the rate of pericyte coverage in the hippocampus of AD mice, we used tomato-lectin to stain endothelial cells, which form the capillaries, and stained pericytes with anti-PDGFR-β. Samples were washed 3 times in TBS containing 0.1% Triton-X (0.1% TBS-X), blocked with 3% normal donkey serum (NDS) diluted in 0.1% TBS-X for 30 min at room temperature, and incubated with specific primary antibodies. The primary antibodies used in this study were anti-GFAP mouse IgG (1:500, Sigma) and anti-platelet-derived growth factor–receptor β (PDGFR-β) goat IgG (1:100, R & D, USA). All antibodies were diluted in 0.1% TBS-X containing 3% NDS. Brain samples were incubated in primary antibodies overnight or for 3 days at 4 °C with shaking. The samples were then washed 3 times with 0.1% TBS-X and incubated with fluorescence-conjugated secondary antibodies for 2 h at room temperature while protected from light. The secondary antibodies used in this study were anti-rabbit IgG donkey Alexa 488 (1:1000, Molecular Probes) and anti-goat IgG donkey cy5 (1:100, Jackson ImmunoResearch). For vascular staining, brain samples were incubated with DyLight 594–conjugated *Lycopersicon esculentum* lectin (1:200, Vector Laboratories, USA) along with secondary antibodies as described by Halliday *et al*.^19^, with some modifications. After incubation, the samples were washed with TBS for 15 min and incubated with DAPI (4, 6-diamidino-2-phenylindole) (1: 10,000 dilution; Sigma) in TBS for 5 min. After a final wash with TBS to remove excess DAPI, the samples were mounted on microscope slides and visualized using a confocal microscope (TCS SP2; Leica, Germany).

### Histochemical senile-plaque staining by X-34

Samples were washed 3 times in TBS and incubated for 30 min with X-34 (1,4-bis(3-carboxy-4-hydroxyphenyl ethyl)-benzene) to stain senile plaques. X-34 is a highly fluorescent dye derived from Congo red that binds specifically to amyloid aggregates and allows detailed visualization of the localization and distribution of senile plaques^5,31,37^. The X-34 solution was synthesized in our laboratory from tetraethyl p-xylylenediphosphonate and 5-formylsalicylic acid. After incubation with X-34, the samples were briefly washed in TBS and incubated for 2 min in a 0.2 g% NaOH/80% ethanol solution. The samples were washed again with TBS for 5 min and incubated with TOTO-3 iodide (Molecular Probes, USA) in TBS for 5 min to stain cell nuclei. The samples were washed again with TBS for 5 min. Images were obtained with a TCS SP2 confocal microscope (Leica, Germany).

### Image processing

Microscopy images were analyzed with ImageJ software. Custom ImageJ plugins were used to automatically establish the overall fluorescence of each image and calculate the percentage of fluorescence, and to automatically detect and analyze the fluorescence data of each image^5^. The region of interest (ROI) used to analyze each image was then inspected manually in debug mode to eliminate false positives and negatives. Confocal-microscopy images of randomly selected fields in the hippocampus (256 µm × 256 µm) were used to count the numbers of pericytes and endothelial cells. To evaluate pericyte coverage, the percentage of the PDGFR-β–labeled area on lectin-labeled endothelial cells was automatically calculated with an ImageJ plugin. Blood-vessel length, the number of pericytes, and the area of pericytes were automatically calculated using ImageJ. Images were analyzed as described by Halliday *et al*.^19^, with some modifications.

### Sandwich ELISA assay

Mice were deeply anesthetized and transcardially perfused with saline to remove blood. The hippocampus was immediately isolated, frozen with liquid nitrogen, and kept at −80 °C until homogenization. The frozen hippocampus was homogenized in ice-cold RIPA buffer containing a protease-inhibitor cocktail (Santa Cruz Biotechnology, Inc. USA) using an ultrasonic homogenizer. After sonication, the samples were incubated for 30 min on ice. The hippocampal homogenates were centrifuged (100,000 g for 1 h at 4 °C), and aliquots of the supernatant were stored at −80 °C until used for ELISAs. Hippocampal amyloid-β (Aβ) 42 levels were determined by sandwich ELISA with the Human/Rat Aβ42 ELISA Kit (Wako, Japan), and IL-1β levels were analyzed with the Mouse IL-1β ELISA Kit (R & D Systems). The hippocampal extracts were appropriately diluted with diluent buffer to fall within the standard range and were applied to ELISA plates. The ELISAs were conducted according to the manufacturers’ instructions. The Aβ42 and IL-1β concentrations were calculated according to a standard curve, and the concentration data were normalized to the total protein concentration. The total protein concentrations of soluble extracts were determined using the BCA Protein Assay Kit (TaKaRa Bio, Japan) according to the manufacturer’s instructions. Data obtained from the hippocampal homogenates were expressed as picomoles of Aβ42 per gram of total protein (pmol/g) or as picograms of cytokine per gram of total protein (pg/mg).

### Statistical analyses

Data were expressed as mean ± SEM. The normality of all data was checked by a chi-square goodness-of-fit test (*p* > 0.05). Data were analyzed by two-way ANOVA, with a Holm-Sidak *post-hoc* test (GraphPad Prism, San Diego). In some experiments, Student’s *t*-test was used. A *p* value less than 0.05 was considered statistically significant.

## Electronic supplementary material

Supplementary Info
