# Supplementary material for: Anserine (beta-alanyl-3-methyl-L-histidine) improves neurovascular-unit dysfunction and spatial memory in aged AβPPswe/PSEN1dE9 Alzheimer’s-model mice
Source: Sci Rep. 2017 Oct 3;7:12571. doi: 10.1038/s41598-017-12785-7 (PMC5626714; doi:10.1038/s41598-017-12785-7)
Supplement: Supplementary file 1 — Supplementary Info [file 41598_2017_12785_MOESM1_ESM.pdf]

Supplementary Information to

Anserine (beta-alanyl-3-methyl-L-histidine) improves neurovascular-unit dysfunction and spatial memory in aged AβPPswe/PSEN1dE9 Alzheimer's-model mice

Jun Kaneko, Akiko Enya, Kota Enomoto, Qiong Ding, and Tatsuhiro Hisatsune

Department of Integrated Biosciences, The University of Tokyo, Kashiwa, Japan

Supplementary Materials & Methods

Animals

B6C3-Tg (APPswe/PSEN1dE9) 85Dbo/J AD-model mice were purchased from Jackson Laboratories (Bar Harbor, Maine, USA), and subsequent generations were bred in our laboratory. In some experiments, we used this transgenic mouse fed a high fat diet (HFD) at 4 months of age5,31. A steady dosage of anserine diluted in autoclaved drinking water was provided, as described in the main Materials and Methods. This treatment was started 2 weeks after the initial feeding with the HFD and continued until the end of the experiments.

Memory tests

Spatial memory performance was assessed by two tests: the MWM and Contextual fear conditioning tests, in independent cohorts of mice. The MWM was performed as described in the main text. In the contextual fear conditioning test, during the last 3 days of HFD, a fear conditioning experiment was carried out as described previously[5,31]. In brief, we used a fear conditioning apparatus made by Med Associates Inc. that consists of a metal-acrylic box connected to a video camera, which enables automated recording and analysis of the experiments.

The novel object location (NOL) task was based on Xiong et al. (2017)[36] with some modifications. In brief, the NOL task was performed across four days: two days for habituation, and the following two days for testing. A white box (45 x 45 x 30 cm) was strategically positioned in a room with various extramaze visual cues. The objects were square and triangular plastic blocks, all of which were cleaned with ethanol after each trial. An overhead camera recorded all of the trials, for the offline scoring and characterization of behavior. During the habituation phase, the mouse was introduced to the center of the empty white box for 10 min each day. On the third day (Day 1 test), two blocks were placed in adjacent corners of the box, 5 cm from the wall. On the fourth day (Day 2 test), the triangular block was displaced to the corner diagonal to the original location (Zone 3: novel location) of the square block (Zone 2: familiar location), and the mouse was again placed in the box and allowed to explore for 4 min. Performance was scored by the amount of time the mouse spent exploring each object, by the SMART video tracking system (Panlab, Barcelona, Spain).

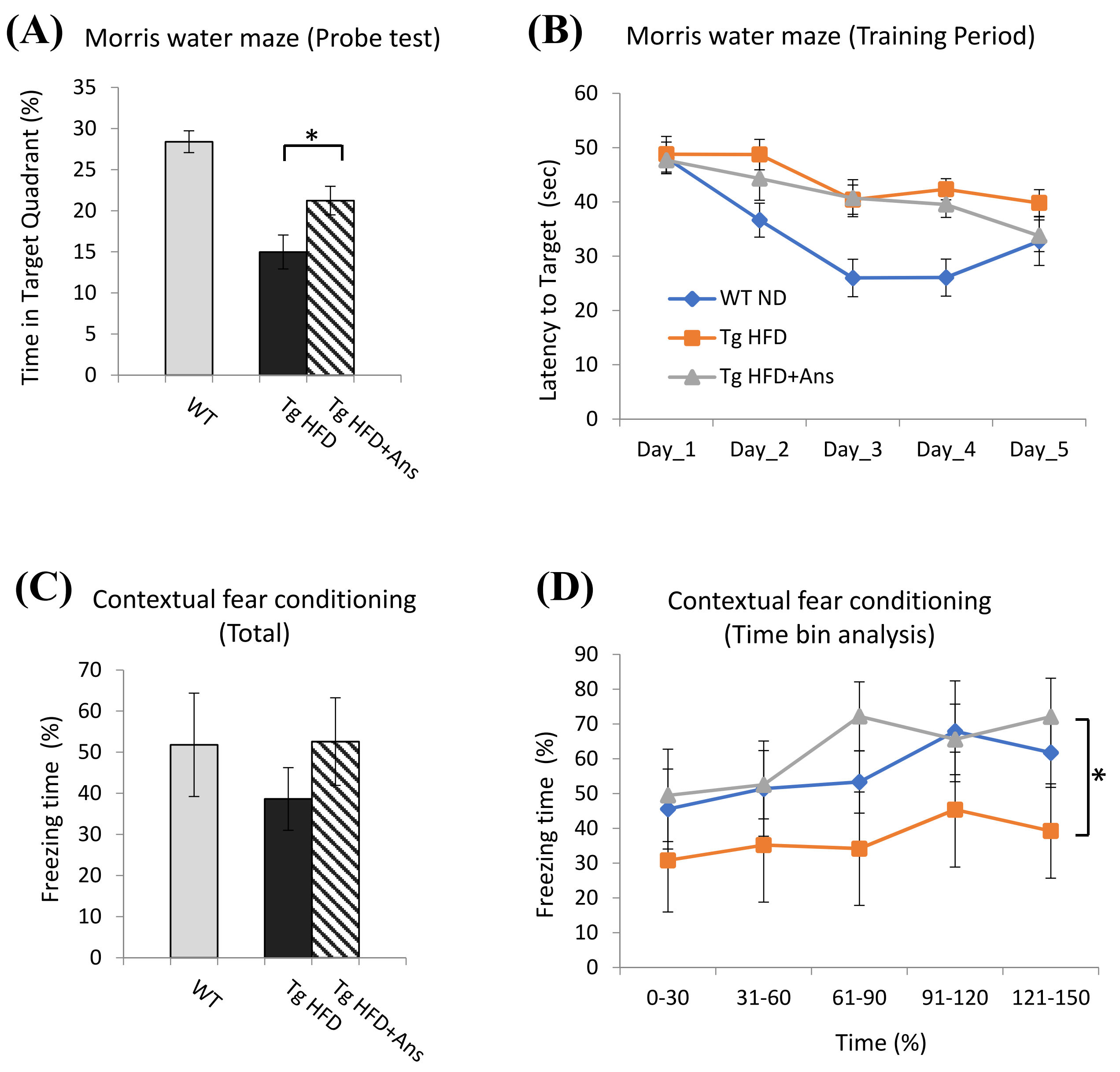

Supplementary Figure 1. Effect of anserine treatment on spatial memory performance tested in two memory tasks: the MWM and contextual fear conditioning tests.

(A) Average time spent in the target quadrant of the probe test of the MWM (each group n=11; wild type [WT], AD transgenic mice fed HFD [Tg HFD], and Tg HFD treated with anserine [Tg HFD+Ans]). Data were analyzed by one-way ANOVA ($F[2,30]=15.01$, $p < 0.0001$). After a Holm-Sidak post-hoc test, we observed a significant difference between Tg HFD and Tg HFD+Ans (t[2.55], * $p < 0.05$). Thus, anserine treatment suppressed the memory deficits in Tg-HFD AD model mice. (B) Escape latencies of each group during training. No significant difference was observed between anserine-treated and untreated AD mice (by one-way ANOVA ($F[2,150]=13.99$, $p < 0.0001$). After a Holm-Sidak post-hoc test, we still did not detect any significant difference between Tg HFD and Tg HFD+Ans ($p > 0.05$). (C) Results of the contextual fear conditioning test (each group n>6). Data were analyzed by one-way ANOVA ($F[2,16]=0.512$, $p = 0.61$). (D) Time block analysis of contextual fear conditioning at 0-150 sec. Data were analyzed by one-way ANOVA ($F[2,80]=4.701$, $p = 0.0117$). After a Holm-Sidak post-hoc test, we detected a significant difference between Tg HFD and Tg HFD+Ans (*$p < 0.05$). These behavior experiments were carried out by an experimenter blinded to the experimental code.

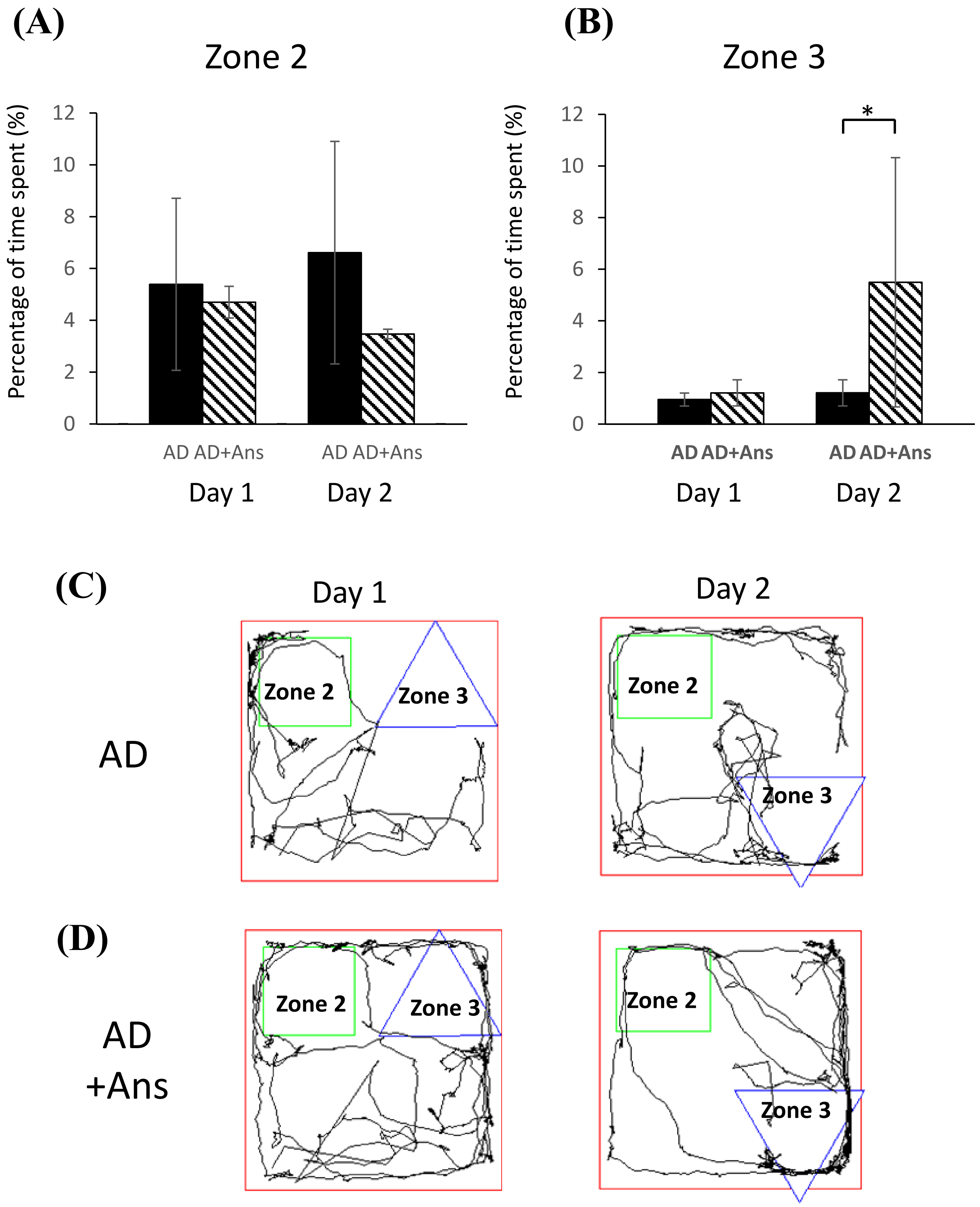

Supplementary Figure 2. Effect of the anserine treatment on memory performance of 18-month-old AD model mice in the novel object location test.

(A) Average time spent in zone 2 (familiar location) by AD and AD+Ans mice. Data were analyzed by repeated two-way ANOVA ($F[1,6]=0.041$, $p > 0.05$). (B) Average time spent in the zone 3 (novel location) by AD and AD+Ans mice. Data were analyzed by repeated two-way ANOVA ($F[1,6]=2.591$). * $p < 0.05$ (post-hoc test). (C, D) Representative trajectory from AD (C) and AD+Ans (D) group mice during the training (Day 1) and test trails (Day 2).
